# Robotic Dual-Console Distal Pancreatectomy: Could it be Considered a Safe Approach and Surgical Teaching even in Pancreatic Surgery? A Retrospective Observational Study Cohort

**DOI:** 10.1007/s00268-021-06216-y

**Published:** 2021-07-24

**Authors:** M. De Pastena, R. Salvia, S. Paiella, G. Deiro, E. Bannone, A. Balduzzi, T. Giuliani, L. Casetti, M. Ramera, C. Filippini, G. Montagnini, L. Landoni, A. Esposito

**Affiliations:** 1 Department of General and Pancreatic Surgery, The Pancreas Institute, University of Verona Hospital Trust, Verona, Italy; 2 University of Verona, Verona, Italy

## Abstract

**Background:**

The study aims to assess the safety and feasibility of the robotic dual-console during a robotic distal pancreatectomy

**Methods:**

The data of the consecutive patients submitted to RDP from 2012 to 2019 at the Verona University were retrieved from a prospectively maintained database. The patients submitted to RDP were divided into the dual-console platform group (DG) and compared to the standard robotic procedure group (SG).

**Results:**

In the study period, 102 robotic distal pancreatectomies were performed, of whom 42 patients (41%) belonged to the DG and 60 patients (59%) to the SG. Higher operation time was recorded in the DG compared to the SG (410 vs. 265 min, *p* < 0.001). The overall conversion rate of the series was 7% (n 7 patients). All the conversions were observed in the SG (*p* = 0.021). No differences in morbidity or pancreatic fistula rate were recorded (*p* > 0.05). No mortality events in the 90th postoperative days were reported in this series.

**Conclusions:**

The robotic dual-console approach for distal pancreatectomy is safe, feasible, and reproducible. The postoperative surgical outcomes are comparable to the standard RDP with the single-console da Vinci Surgical System®. This surgical technique can widely and safely improve the robotic surgical training program.

## Introduction

The minimally invasive approach is obtaining wide popularity in pancreatic surgery, especially for the resections of the left part of the pancreas. Even if available in the literature only non-randomized studies comparing open resection with minimally invasive approaches, several benefits are reported about the latter's use, including less blood loss and shorter hospital stay [1–3]. Remarkably, the minimally invasive approach to the distal pancreatic lesions is widely accepted when it is feasible as the gold standard for benign and uncertain behavior tumors [4, 5].

Despite its potential benefits, the technical limitations of conventional laparoscopy, such as rigid, non-articulated instruments and uncomfortable ergonomics, could preclude the diffusion and the implementation of a minimally invasive approach in pancreatic surgery. The introduction of robotic technology affords the surgeon to overcome the limitations of the conventional minimally invasive approach. Indeed, the da Vinci system (Intuitive Surgical, Sunnyvale, CA, USA) provided a better and magnified visualization of the surgical field with the 3D resolution, reduced natural tremors, increased surgical precision, and dexterity the introduction of the intraabdominal articulating instruments [6]. All these surgical and technical benefits seem to positively influence the clinical outcomes and reduce postoperative complications and hospitalization [7, 8].

However, robot surgery emphasizes the single surgeon figure, which usually performs the procedure alone in the console. Unlike the laparoscopic or the open approach, the robotic procedure lost part of the benefits obtained from the assistant surgeons. This issue could be partially solved by the introduction of the da Vinci system dual-console. The use of the dual-console has shown advantages in reducing the learning curve and surgical training during different surgery [9, 10]. To our knowledge, no data are reported in the literature regarding the use of the robotic dual-console in pancreatic surgery. Furthermore, no data are available to use, routinely, the dual-console, reproducing the steps and the movements of laparoscopic or open approach during pancreatic resection by expert surgeons, not only during the training program.

The study aims to assess the safety and feasibility of the robotic dual-console during a Robotic distal pancreatectomy (RDP).

## Materials and methods

The study was performed according to the Strengthening the reporting of observational studies in epidemiology (STROBE) and Strengthening the reporting of cohort studies in surgery (STROCSS) guidelines [11, 12]. The Institutional Review Board’s approval for data collection and analysis was obtained. Written informed consent was collected.

The consecutive patients submitted to RDP from 2012–2019 at the Verona University were retrieved from a prospectively maintained database.

Each patient was submitted preoperatively to a Contrast-enhanced CT scan of the abdomen. All cases were preoperatively reviewed at a dedicated institutional surgical meeting, where the decision to perform a minimally invasive procedure was undertaken among staff surgeons. The Da Vinci Surgical System® Xi dual-console platform (Intuitive Surgical, Sunnyvale, CA) was used to perform the RDP from 2018. Indications for the use of the robot or the type of procedure were based on the availability of the Da Vinci Surgical System® and the surgeon's judgment, as previously described [13, 14]. Briefly, indications for a minimally invasive approach were benign or pre-malignant lesions smaller than 10 cm or, for malignancies, tumors without evidence of major vessel involvement. Additional resections, beyond cholecystectomy, adrenalectomy, or wedge resection of the stomach, were an exclusion criterion.

### Data collection and definitions

Demographic and baseline characteristics consisted of sex, age, body mass index (BMI, kg/m^2^), American society of anesthesiologists (ASA) physical status, and previous abdominal surgery.

The intraoperative data were composed of the type of procedure, conversion rate, operative time (minute), blood loss (milliliter), and pancreatic stump management (stapler or ultrasonic device).

All 90 day postoperative complications were scored and classified with the Clavien-Dindo system and the Comprehensive complication index [15]. The major complications as Clavien-Dindo grade III or higher. The postoperative pancreatic fistula was defined by the updated definition [16].

The length of stay (days), 90 day reoperation, readmission, and mortality rates were recorded. The final pathology, tumor size (mm), and the number of lymph nodes harvested were also collected.

### Surgical procedure

During the study period, the technique did not change regarding the steps of the procedure, the transection technique adopted, and the difficulty level. Briefly, the robotic docking was performed at the patient’s head, which was in a supine position, at least 15 cm from the operating table. The open technique created a pneumoperitoneum at the umbilicus with 12 mmHg CO_2_ pressure. Based on the different types of robots, the trocars were placed under visual control: the optical port at the umbilicus, two in the left hypochondrium, and one in the right hypochondrium. The assistant operative 12 mm port was positioned in a lower position between the umbilical and the first left port. The latter was usually used for the ultrasonic devices (HARMONIC FOCUS or ACE®; Johnson & Johnson Medical, Ethicon, Somerville, NJ, USA), scissors, clip applicator, suction, and the eventual transection of the pancreas by stapler device. Robotic instruments, such as a grasper, bipolar coagulation, and monopolar hook were routinely used. Considering the indication for RDP, the pancreatic transection level and the splenic preservation were tailored. The intraoperative ultrasound was used to assist the surgical team in the selection of organ sparing procedures. The management of the pancreatic stump was reported previously [17], consisting of a stapler reinforced with a PGA felt (NEOVEIL® Endo GIA™ Reinforced Reload with Tri-Staple™ Technology 60 mm; COVIDIEN, North Haven, CT, USA), or an ultrasonic dissector (HARMONIC FOCUS or ACE®; Johnson & Johnson Medical, Ethicon, Somerville, NJ, USA). No additional sutures or patches were added during both techniques. The surgical field was drained by a surgical tube placed proximal to the pancreatic remnant. The postoperative drain management was based on the policy of early drain removed previously published [18].

### Minimally invasive surgeons’ expertise and training strategy adopted

The same two surgeons always performed the surgical procedures, which already had completed the learning curve in laparoscopic distal pancreatectomy. According to Napoli et al. [19], the leading senior surgeon completed the learning curve of RDP during the standard technique phase, while the trainee achieved this result during the dual-console phase. The assistant surgeons at the bedside were always experienced pancreatic surgeons that already completed the learning curve in laparoscopic distal pancreatectomy.

The Verona robotic training program was reserved for the surgeons that completed the laparoscopic distal pancreatectomy learning curve. The training program was divided into three different steps. Initially, all the trainees received the detailed technique description. The latter was composed of a checklist of the surgical instruments and a detailed procedure description and tips and tricks to prevent and solve potential intraoperative adverse events. Step one included the video training. Video training revised the entire procedure, and surgical tips and tricks were discussed with the senior surgeon. Step two was composed of the simulator training and cadaver lab. The robotic training followed the Intuitive Surgical recommendations and the official DaVinci surgical training. The third step was on-site proctoring by the senior surgeon.

### Statistical analysis

Continuous variables were presented as means and standard deviation, or median and interquartile range, when pertinent. Student’s t and Mann–Whitney U tests were used to compare continuous variables. Nonparametric tests were used when appropriate. Comparative analysis between groups was conducted using Fisher’s exact tests for categorical variables. A *p* value < 0.05 was considered statistically significant (two-tailed). Data were analyzed using Statistical Package for the Social Sciences 25.0 (IBM Corp., Armonk, NY, USA).

## Results

**Fig. 1:**
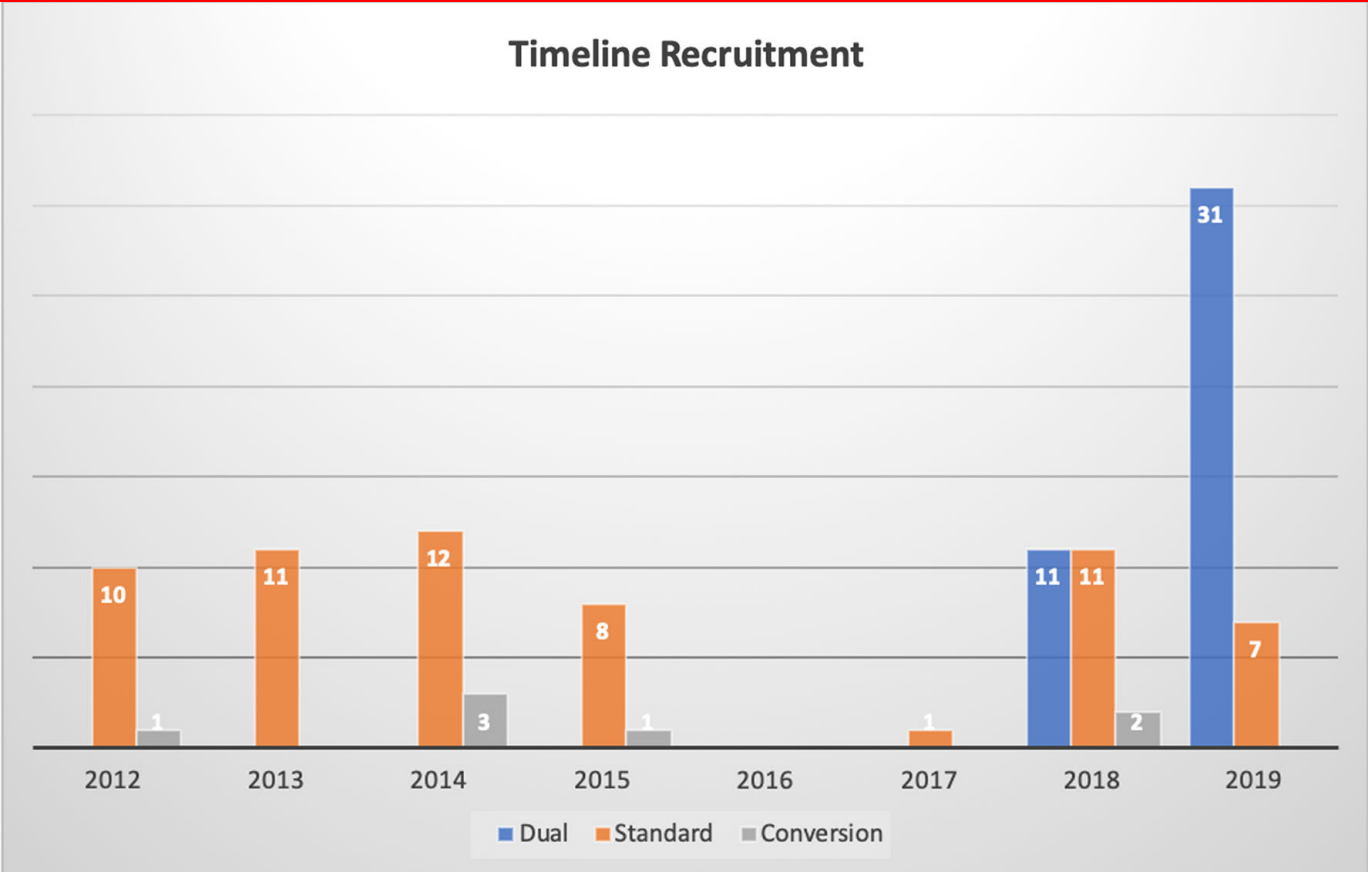
Timeline recruitment

In the study period, 102 robotic distal pancreatectomies were performed, of whom 42 patients (41%) belonged to the Dual-console technique group (DG) and 60 patients (59%) to the Standard procedure (SG). Demographic, intraoperative, and pathological data are shown in table 1. The baseline characteristics of the patients were balanced between groups. Higher operation time was recorded in the DG compared to the SG (410 vs. 265 min, *p *< 0.001). The overall conversion rate of the series was 7% (n 7 patients). All conversions were observed in the SG (*p* = 0.021). The approach to robotic distal pancreatectomy over time with the conversion rate is reported in Fig. 1. Moreover, different management of the pancreatic stump was recorded (*p* < 0.001). The ultrasonic scalpel was the most common device used in the SG (80%). Otherwise, the stapler was frequently used during the dual-console technique (55%). Notably, the two patients' pancreatic transection was performed by the knife associated with the stump oversewn.

**Table 1:** Demographic, intraoperative, and pathological data

Study population N = 102				
	Total n(%)	Dual-console group 42 (41%)	Standard group 60 (59%)	*p*-value
Age (years, DS)	51 ± 16	51 ± 16	51 ± 15	0.810
Sex (Female)	73 (72%)	28 (67%)	45 (75%)	0.243
BMI (Kg/m^2^, IQR)	23 [22–27]	23 [20–29]	23 [22–27]	0.603
Previous abdominal surgery	42 (41%)	20 (48%)	22 (37%)	0.184
ASA score I-II	94 (92%)	37 (88%)	57 (95%)	0.183
Spleen preservation	35 (34%)	17 (41%)	18 (30%)	0.188
Conversion	7 (7%)	0 (0%)	7 (12%)	**0.021**
Pancreatic transection level				0.568
GDA	2 (2%)	0 (0%)	2 (3%)	
Neck	58 (57%)	25 (60%)	33 (56%)	
Distal	42 (41%)	17 (40%)	25 (41%)	
Management stump				** < 0.001**
Stapler	33 (32%)	23 (55%)	10 (17%)	
Ultrasonic scalpel	67 (66%)	19 (45%)	48 (80%)	
Duration of surgery (minutes, IQR)	337 [260–401]	410 [330–480]	265 [230–330]	** < 0.001**
EBL (cc, IQR)	150 [100–250]	150 [50–225]	100 [100–275]	0.811
Pathology, No. (%)				0.687
PDAC	10 (10%)	5 (12%)	5 (8%)	
Cystic lesions	28 (27%)	9 (21%)	19 (32%)	
pNET	42 (41%)	18 (43%)	24 (40%)	
Other	22 (22%)	10 (24%)	12 (20%)	
Tumor size (mm, DS)	31±22	30 ± 20	32 ± 23	0.661
Harvest lymph nodes (DS)	15 ± 12	15 ± 11	14 ± 12	0.666

Statistically significant values (*p* < 0.05) are given in bold

ASA: American society of anesthesiology; BMI: Body mass index; GDA: Gastroduodenal artery; MCN: Mucinous cystic neoplasm; pNET: Pancreatic neuroendocrine tumor; SPT: Solid pseudopapillary tumor

Regarding the final pathology, the groups were homogenous (*p* = 0.687). No differences were found in the mean tumor size (30 vs. 32 mm) and harvest lymph nodes (15 vs. 14) (*p* = 0.661 and* p* = 0.666, respectively).

Postoperative outcomes were outlined in table 2. The overall morbidity was 43% (n 44 patients) without difference between groups (41% vs. 45%, *p* = 0.402). The postoperative pancreatic fistula occurred in 29 patients (28%), similar between the two groups (31% vs. 27%, *p* = 0.400). Two grade C fistulae were detected in the SG. Five patients required a laparoscopic surgical revision. No statistical differences between groups were recorded (5% vs. 12%, *p* = 0.199). No mortality events in the 90th postoperative days were reported in this series.

**Table 2:** Postoperative data

Study population N = 102				
	Total n(%)	Dual-console group 42 (41%)	Standard group 60 (59%)	*p*-value
Overall morbidity	44 (43%)	17 (41%)	27 (45%)	0.402
Major complications	10 (10%)	2 (5%)	8 (13%)	0.136
CCI	10 ± 14	9 ± 14	11 ± 15	0.631
POPF	29 (28%)	13 (31%)	16 (27%)	0.400
PPH	9 (9%)	2 (5%)	7 (12%)	0.199
Length of stay (days, IQR)	7 [6–10]	7 [7–10]	7 [6–10]	0.618
Reoperation	8 (8%)	1 (2%)	7 (12%)	0.086
Readmission	12 (12%)	2 (5%)	10 (17%)	0.060

CCI: Comprehensive complication index; POPF: Postoperative pancreatic fistula; PPH: Post pancreatectomy hemorrhage

## Discussion

The robotic dual-console approach for distal pancreatectomy is safe and feasible. Even if there is an initial increase in the operation time, it does not negatively impact the clinical course in terms of postoperative outcomes. Indeed, the mortality nihil and no conversion were performed. The use of the dual-console da Vinci Surgical System® is not inferior compared to the standard RDP in a high volume of robot-assisted pancreatic surgery.

Minimally invasive robotic pancreatic surgery continues to gain popularity compared to laparoscopy due to improved patient outcomes, such as reduced hospital stay and faster recovery time [3]. RDP's learning process is still under investigation, varying from 10 to 40 cases to complete the learning curve [19–21]. Within a niche specialty, such as robotic pancreatic surgery, several years may be necessary to make the surgeon complete an RDP safely. However, rather than the time needed to attain it, it is fundamental to do it safely.

Trainees of robotic surgery should learn by doing rather than by observing [22]. Simulators or training platforms are helpful to familiarize with the technique and memorize all the procedures' steps. However, the introduction of the dual-console da Vinci Si Surgical System® allows an active and, at the same time, controlled training [23]. Indeed, it has been widely accepted that active trainee attendance during surgery on the robotic console is vital to improving the robotic curriculum [24].

The traditional surgical teaching model adopted for open surgery, “see one, do one, teach one”, is hard to apply to robotic surgery, where, instead, the gradual transition of autonomy from attending surgeon to the trainee may be safely and quickly possible using the dual-console technique. The second console's introduction allows controlled and safe teaching, reproducing the open surgery steps and differing from laparoscopy. Furthermore, during the robotic procedure, the surgeon can learn more complex procedures step by step, offering the possibility to the proctor to activate, modify or improve the surgical act, like open surgery. The traditional surgical teaching model adopted for open surgery can be reviewed for robotic surgery in “see one, do side by side one, teach one.”

This approach has already been investigated and validated in different abdominal surgeries and specialties [9, 25, 26]. These studies demonstrated that the dual-console robotic system could overcome defects of laparoscopic surgical training. The real essence of laparoscopic surgery is usually associated with an impression of distance and dissociation between the proctor and the trainee. During a laparoscopic procedure, the classical ‘‘4-handed’’ technique is usually uncomfortable due to the interference and conflict that could be generated by the laparoscopic instruments and camera and the surgeons. Furthermore, the continuous switching from the first operator function and the camera between proctor and trainee is a protracted, slow, and complicated movement during surgery. The exchange of the camera, which causes a temporary loss of the surgical vision, resetting the operative field to the original status, results in increased operative time, perseverance, and sometimes tolerance from the proctor. Additionally, the proctor's attention cannot always have the procedure under control increases anxiety and stress. All these issues may be solved by introducing the robotic dual-console technique.

The study has some limitations that may prevent it from being generalized. First, the research was conducted on a retrospective basis. Therefore, the selection bias may be burdensome in choosing the type of surgery performed and even the robotic-assisted approach itself. Second, the sample size is limited, reducing the power of the statistical analysis. Third, the dual-console approach was introduced recently. This could possibly generate a selection bias. Fourth, the specific cost of this technology could be a limit to its diffusion. Has been widely described that the robotic technology has still high and important crude costs and its economic impact is debated yet [13]. Even if the literature reported promising surgical outcomes, the expenses of the robot application are reducing the possibility to perform surgical procedure and implement the surgical robotic training program. However, the diffusion of robotic technology could implement and improve this surgical approach, encouraging the introduction of the dual-console in a standardized training program.

The study results demonstrated the safety and the feasibility of the dual-console approach in pancreatic surgery. Future studies should prove it routinely useful.

## Conclusion

The robotic dual-console approach for distal pancreatectomy is safe, feasible, and reproducible. The postoperative surgical outcomes are comparable to the standard RDP with the single-console da Vinci Surgical System®. This surgical technique can widely improve the robotic surgical training program. Further studies are required to standardize the surgical technique and the robotic surgical training program.
